# P-744. Vancomycin De-Escalation Utilizing Methicillin Resistant Staphylococcus aureus Nasal Swabs in the Setting of Skin and Soft Tissue Infections

**DOI:** 10.1093/ofid/ofaf695.955

**Published:** 2026-01-11

**Authors:** Abriana M Palumbo, Satwinder S Kaur, David T Adams

**Affiliations:** Texas Health Harris Methodist Ft Worth, Euless, TX; Texas Health Harris Methodist Hospital Fort Worth, Fort Worth, Texas; Texas Health Harris Methodist Hospital Fort Worth, Fort Worth, Texas

## Abstract

**Background:**

MRSA PCRs are commonly used for de-escalation of vancomycin when treating patients with respiratory tract infections. Despite less robust data supporting the performance of this test for skin and soft tissue infections (SSTIs), prior retrospective data has demonstrated a high negative predictive value (NPV). Recently at Texas Health Harris Methodist Hospital Fort Worth, an internal pharmacist-driven protocol was implemented where MRSA PCRs are ordered when vancomycin is initiated for SSTIs. The purpose of this study was to determine the impact of utilizing MRSA PCRs on vancomycin days of therapy for SSTIs.

**Methods:**

A single center, retrospective, observational cohort study of patients receiving vancomycin with indications for cellulitis, abscess, or diabetic foot infection. Patients without a MRSA PCR were included in the pre-group and patients with a MRSA PCR after protocol implementation were included in the post-group. The primary outcome was days of vancomycin therapy. Secondary outcomes evaluated were number of vancomycin levels obtained, in-hospital mortality or 30-day readmission due to the same SSTI, and rate of acute kidney injury.

**Results:**

188 patients were included with 94 patients in each group. Baseline characteristics were similar between both groups except for ethnicity and weight. After protocol implementation, a statistically significant reduction in vancomycin days of therapy was observed in the post-group (3 days vs 4 days, *p* = 0.0139). No statistically significant difference was observed for in-hospital mortality or 30-day hospital readmission due to an SSTI. Within the post-group, 80.9% (76/94) of MRSA PCRs resulted negative and 56.6% (43/76) of those negative results were utilized to facilitate de-escalation off vancomycin therapy. The MRSA PCR NPV was 98.7% (75/76) in our cohort.

**Conclusion:**

Based on the results of this study, the utilization of MRSA PCRs to facilitate de-escalation of vancomycin in SSTIs resulted in fewer days on vancomycin without seeing an increase in re-admission or mortality. While prospective data would be beneficial, our study contributes to the growing body of evidence supporting the use of MRSA PCRs as a safe and effective antimicrobial stewardship tool to help reduce unnecessary antibiotic exposure for patients with SSTIs.

**Disclosures:**

All Authors: No reported disclosures

